# Design and Simulation of Microbolometer with Dual Cavity for High Figure of Merits

**DOI:** 10.3390/mi14050948

**Published:** 2023-04-27

**Authors:** Kevin O. Díaz Aponte, Yanan Xu, Mukti Rana

**Affiliations:** 1 Division of Physics, Engineering, Mathematics and Computer Sciences, Delaware State University, Dover, DE 19901, USAyxu@desu.edu (Y.X.); 2 Optical Center for Applied Research, Delaware State University, Dover, DE 19901, USA

**Keywords:** thermal sensors, microbolometer, infrared detector

## Abstract

The rapid expansion of the applications of infrared (IR) sensing in the commercial market has driven the need to develop new materials and detector designs for enhanced performance. In this work, we describe the design of a microbolometer that uses two cavities to suspend two layers (sensing and absorber). Here, we implemented the finite element method (FEM) from COMSOL Multiphysics to design the microbolometer. We varied the layout, thickness, and dimensions (width and length) of different layers one at a time to study the heat transfer effect for obtaining the maximum figure of merit. This work reports the design, simulation, and performance analysis of the figure of merit of a microbolometer that uses Ge_x_Si_y_Sn_z_O_r_ thin films as the sensing layer. From our design, we obtained an effective thermal conductance of $1.0135\times 10^{-7}\;W/K$, a time constant of 11 ms, responsivity of $5.040\times 10^{5}\;V/W$, and detectivity of $9.357\times 10^{7}\;cm-Hz^{1/2}/W$ considering a 2 μA bias current.

## 1. Introduction

Thermal detectors’ operation is related to the absorption of incident radiation, among others. This absorption is determined by the spectral-dependent surface absorptance of the detector or absorber material. According to Planck’s law, any object whose temperature is greater than 0 K radiates at thermal equilibrium, and the spectral radiance of this electromagnetic radiation can be expressed by Equation (1).

(1) $$M_{e}\left(\lambda ,T\right)=\frac{2\pi hc^{2}}{\lambda^{5}}\frac{1}{e^{\frac{hc}{\lambda k_{b}T}}-1}$$

Here, $M_{e}$ = spectral radiant existence, T = temperature, *λ* = wavelength, h = Plank’s constant, c = speed of light in the propagating medium, and $k_{b}$ = Boltzmann’s constant. When infrared (IR) radiation from an object is absorbed by a detector material, the temperature of the material changes and a measurable change in electrical signal is produced. This change in the electrical properties of the material can be measured. For an object at a temperature of 300 K, the peak emittance occurs around 9.5 µm of wavelength, according to Planck’s law [1]. So, Planck’s law gives us the feasibility of using a detector operating at room temperature. The typical spectral response for thermal detectors is in the IR region (700 nm–1 mm) [2,3].

Microbolometers are thermal detectors in which the resistance changes in the sensing layer because of the absorption of irradiation energy [4,5]. This change in resistance is associated with the temperature coefficient of resistance (TCR) of the material, which is defined by Equation (2).

(2) $$\alpha =\frac{dR}{dT}$$

where *R* is the resistance of the material, which has a TCR of α when a temperature change of *dT* yields a resistance change of *dR*. Both the TCR and resistivity are material properties, and materials with a higher TCR would yield microbolometers with higher figures of merit such as responsivity and detectivity. Microbolometers are widely used in many applications, which include defense and security, surveillance, autonomous driving, and many others. As no cooling is required, these detectors are lightweight as compared to their counterparts, such as photon detectors, making them easily portable. The absorption of IR radiation in the sensing material creates a very small change in temperature. Because of this, it is very important to have good thermal isolation so the absorbed radiation can be prevented. Excellent thermal isolation in combination with a material with a high TCR will exhibit a microbolometer with higher figures of merit.

The linear equation relating resistance and temperature change is expressed by Equation (3).

(3) $$R=R_{0}(1+\alpha ∆T)$$

where R is the final resistance of the material when there is a temperature change of ∆T, and $R_{0}$ is the initial resistance at room temperature. TCR depends on the properties of the material. In the case of semiconductors, the TCR is negative and has an exponential temperature dependence. Because of the negative TCR, the decrease in temperature increases the current flow through the sensing layer, and this could be burned out if we do not have a good model.

The temperature change of a body, ∆T, subjected to IR radiation is defined by Equation (4).

(4) $$\left|∆T\right|=\frac{\varepsilon \varphi }{K_{eff}\sqrt{1+\omega^{2}{\tau_{th}}^{2}}}$$

The temperature gradient ∆T is defined as the difference in temperature between the detector and the substrate. This temperature change will depend on the heat flux φ, which is irradiated by the sensor, which has an emissivity of ε. Here, ω that is the angular frequency related to the irradiant energy that falls on the sensor, $\tau_{th}$ is the thermal time constant, and $K_{eff}$ is the effective thermal conductivity of the microbolometer.

Besides TCR, there are two important figures of merit that are used to analyze the performance of a microbolometer. These are - responsivity and detectivity. Primarily, the values of these figures of merit indicate the fundamental performance limits of thermal detectors.

The voltage responsivity, $R_{v}$ of a microbolometer is defined as the amount of output power obtained per unit of radiant optical power input and can be expressed by Equation (5).

(5) $$R_{v}=\frac{\eta \alpha RI_{b}}{K_{eff}\sqrt{1+\omega^{2}\tau_{th}^{2}}}$$

where $I_{b}$ is the bias current.

Another important figure of merit detectivity is the area normalized signal-to-noise ratio, which is expressed by Equation (6).

(6) $$D*=\frac{R_{v}\sqrt{A_{d}∆f}}{v_{n}}$$

where $R_{v}$ is the voltage responsivity, *A_d_* is the detector area, ∆f is the noise-equivalent bandwidth, and $v_{n}$ is the total noise.

$K_{eff}$ is related to the heat transfer in solid by conduction ($K_{cond}$), convection ($K_{conv}$) and radiation ($K_{rad}$). For a microbolometer operating in vacuum, the bolometer must be enclosed in vacuum to increase the performance so that we can ignore the convection, $K_{conv}$. Therefore, we can calculate this value by considering Equation (7).

(7) $$K_{eff}=K_{cond}+K_{rad}$$

A microbolometer is composed of different layers and materials with different thermal conductivities. Therefore, we need to consider these different layers and dimensions between the irradiation and the heat sink to calculate the value of $K_{cond}$. For a microbolometer operating in vacuum, $K_{rad}$ is related to how much of the irradiation energy is absorbed by the microbolometer. The value of this could be calculated by Equation (8).

(8) $$K_{rad}=4\sigma_{e}\varepsilon A_{d}T^{3}$$

where $\sigma_{e}$ is the Stefan–Boltzmann constant. We can say that this is the only way by which heat can be conducted between the detector and the surrounding area when a microbolometer is operating in vacuum. Other than the detector area, other parameters that affect the value of $K_{rad}$ are dependent on the material properties.

The thermal conductance of a microbolometer depends on the structure and volume of the arms. A well-designed arm can perfectly balance the heat conduction towards the heat sink and the thermal time constant of the device. The value of thermal conductance has a direct effect on the responsivity of the device and is dependent on the $K_{cond}$. This value can be calculated using Equation (9).

(9) $$K_{cond}=k_{arm}\frac{A_{arm}}{L_{arm}}=\sum_{i}k_{i}\frac{A_{i}}{L_{i}}$$

where $k_{arm}$ is the thermal conductivity of the arm, $A_{arm}$ is the cross-sectional area of the arm, and $L_{arm}$ is the length of the arm of the microbolometer. This equation can be applied to i numbers of “layers” that make up the arms to calculate the effective thermal conductance. In our model, the thermal conductance depends on two arms made of titanium. The resulting effective thermal conductance can be expressed by Equation (10).

(10) $$K_{cond}=2\left(k_{Ti}\frac{A_{Ti}}{L_{Ti}}\right)$$

The thermal time constant ($\tau_{th}$) is equal to the heat capacity over the thermal conductance. This value represents the time required by the sensor to reach 63% of its possible maximum temperature.

The effect of the heat capacity of the model is considered to evaluate the performance of the device. To calculate the value of the heat capacity of the model, we use Equation (11).

(11) $$C_{tot}=\sum_{i}V_{i}C_{i}\rho_{i}$$

where C, ρ, and V are the specific heat capacity, density, and volume, respectively, of the materials that constitute the detective membrane. Therefore, we could express Equation (12).

(12) $$C_{tot}=V_{NiCr}C_{NiCr}\rho_{NiCr}+V_{Ti}C_{Ti}\rho_{Ti}+V_{GeSiSnO}C_{GeSiSnO}\rho_{GeSiSnO}$$

Knowing these two values, we can calculate the value of the thermal time constant of the model using Equation (13).

(13) $$\tau_{th}=\frac{C_{tot}}{K_{eff}}$$

From Equation (4), it can be seen that lower values of $\tau_{th}$ and $K_{eff}$ would yield higher ∆T, which would increase the detectivity and responsivity. While TCR is a purely material property, responsivity and detectivity of a microbolometer can be increased by lowering $\tau_{th}$ and $K_{eff}$ values through the design. In this work, we studied the change in temperature in the sensing layer as a function of time to study the thermal time response when we modified the dimensions of various layers to obtain higher figures of merit of a microbolometer.

To this day, various microbolometer designs have been mentioned and implemented [6,7,8,9,10,11,12,13,14,15,16]. Each model the scientific and engineering community presents seeks to improve its capabilities. They have been focused on developing the fabrication of dense pixel arrays to improve image quality, leaving aside the sensitivity and detectivity of the pixel. Recently, the discussion of improving these two parameters has been revived with the emergence of new semiconductor alloys and nanomaterials. In this work, we designed a dual-cavity microbolometer that used alloys of germanium, silicon oxygen, and tin (Ge_x_Si_y_Sn_z_O_r_) as the sensing layer [17]. This work reports the design, simulation, and performance analysis of the figure of merit of a microbolometer that used Ge_36_Si_0.04_Sn_11_O_43_ thin films as the sensing layer.

## 2. Materials and Methods

We used the finite element method (FEM) utilized by COMSOL Multiphysics software to design the microbolometer and construct the three-dimensional model. We also used the same software to determine the figures of merit of the microbolometer.

COMSOL provides an integrated development environment (IDE) that combines a model builder in 2D and 3D environments and workflows to mix more than one physics phenomenon [18]. In COMSOL, we can couple many physical phenomena to the model we built to obtain the responses of the devices. COMSOL provides heat transfer with surface-to-surface radiation (HTSSR) Multiphysics coupling to model this phenomenon [19]. When we added this module to the model builder, it automatically added the heat transfer at the solid interface (HTSI) and surface-to-surface radiation interface (SSRI) [20]. These two physics are coupled. This combination provides the governing equations and boundary condition nodes necessary to simulate the heat transfer by irradiation using the blackbody theory. Moreover, we used the electric current interface (ECI) to study the effect of heat change on the resistance of the sensing layer [21].

We studied the heat transfer process in the microbolometer model and observed how this phenomenon affected the electrical resistance of the microbolometer. One of our objectives is to see the effect of applied heat flux on the temperature change of the sensing material and thereby the change in the resistance of the sensing material. The implementation of this computational model helped us understand the thermal heat distribution and electrical response of the microbolometer and determine the figures of merit in detail [22].

**Figure 1:**
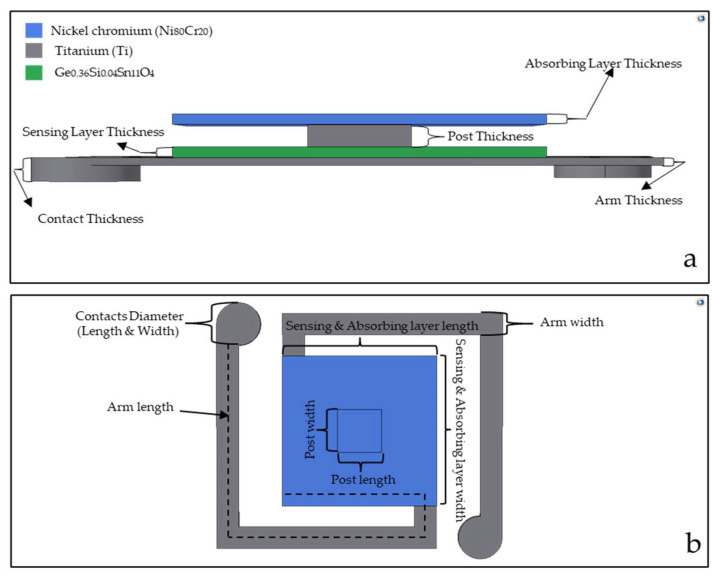
(**a**) Side and (**b**) top view of bolometer having dual cavity.

Our current work is based on the design and simulation of a microbolometer using a double sacrificial layer. Figure 1a shows the side view, while Figure 1b shows the top view of the microbolometer we designed using two cavities. In this design, two overhanging layers were built on top of the substrate using sacrificial layers. The first layer from the bottom is made of the sensing material, while the top layer represents the absorber. Here, titanium (Ti) was used as the electrode arm layer, while nickel–chromium (Ni_80_Cr_20_) was used as the absorber layer.

In Table 1, we show the properties of the nickel–chromium (Nr_80_Cr_20_) alloy used to build the absorbing layer [23]. Titanium (Ti) was used to build the post, arms, and contacts [24], and germanium–silicon–tin–oxide (Ge_36_Si_0_._04_Sn_11_O_43_) to build the sensing layer [17]. The resistivity parameter used in the simulation for the sensing layer was measured using a Linseis thin film analyzer. The measured electrical resistivity value using a four-point probe was $293\times 10^{-3}$ Ωm. Using this value, we computed the device’s resistance using COMSOL. At room temperature, the resistance was found to be 1.049 MΩ.

It was assumed that the device’s initial temperature was 298 K, and a heat flux of 50 $W/m^{2}$ was applied to the device to see the effect of irradiation on the sensing layer. We varied the thickness, dimension, and geometry of different layers to see the parametric effect of them on the device’s performance. The design was optimized to see the effect of it on the temperature rise of the sensing material. Table 2 shows the dimensions of different layers along with the materials we used to optimize the performance of the device. The reason for this is that these parameters have a direct relationship with thermal conductance and the response time of the device. The purpose of varying these parameters is to find a balance between heat capacity and response time.

For the study of heat transfer, we used 1000 ms as the study time in steps of 1 ms for our simulation. This study computes the change in temperature in the microbolometer in this time frame for different arm widths and thicknesses.

One of the biggest challenges of the computer simulation was finding the correct values between the meshing parameters and the “fully couplet” setting values. Many times, the simulation diverges because of an inconsistency in the tolerance value. Therefore, we set the values in the “fully couplet setting” section to improve the convergence. We calculated the responsivity, detectivity, and NEP of the device using equations [11,12,13].

To calculate the thermal time constant of the model, we used the analytical Equation (14); where $T_{0}$ is the initial temperature and $∆T_{max}$ is the maximum change in temperature.

(14) $$T_{63\%}=T_{0}+\left(0.63\cdot {∆T}_{max}\right)$$

## 3. Results and Discussion

The results branch in the COMSOL Multiphysics model tree contains tools for postprocessing and analyzing the simulation results, including visualizations and data analysis. In our case, COMSOL produced a visual model of heat transfer and the electrical potential. We determined the heat produced at the sensing layer and the change in resistance. Eventually, the simulation results would yield the values of various device parameters, such as voltage responsivity and detectivity.

### 3.1. Heat Transfer in the Model

**Figure 2:**
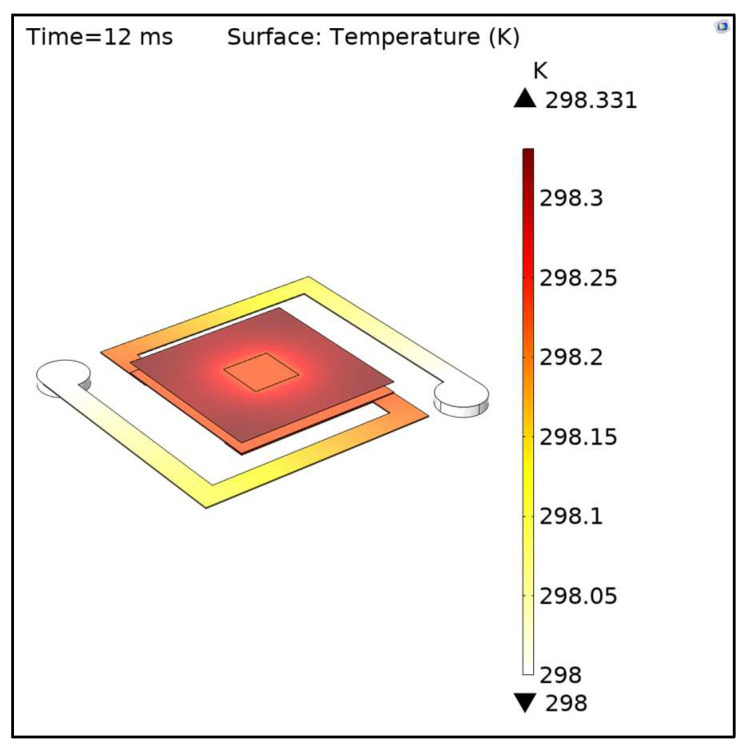
Visualization of the heat transfer in solid after 12 ms.

After the simulation, the software displayed a visualization of the heat transferred into the device. In Figure 2, we can see the heat distribution in the model after 12 ms. Each model’s result will differ because of the difference in arm widths and thickness variations. This difference is because the temperature rise is proportional to the thermal path, which in this case is related to the arm and contact layer’s dimensions.

### 3.2. Heat Transfer in the Sensing Layer

**Figure 3:**
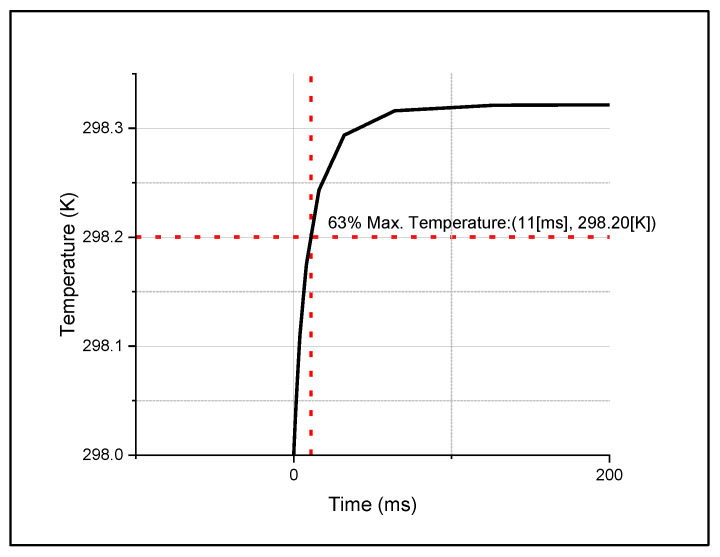
Temperature rises in microbolometer with time.

While performing the simulation, we found that the width and thickness of the electrode arms significantly impacted the microbolometer’s performance. Therefore, we focused on this parameter to compute the temperature change at the sensing layer. To perform this analysis, we considered the temperature change from the initial value to 63% of the maximum temperature, as shown in Figure 3.

**Figure 4:**
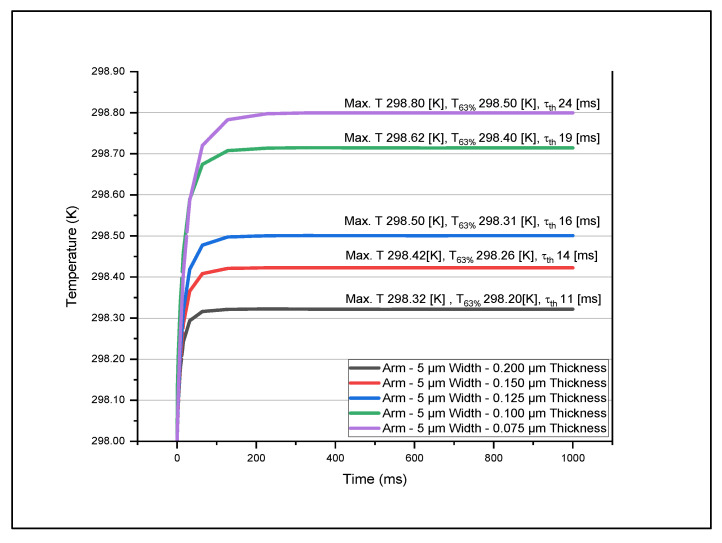
Variations in temperature with time at the sensing layer for different thicknesses with arm width kept fixed at 5 μm.

**Figure 5:**
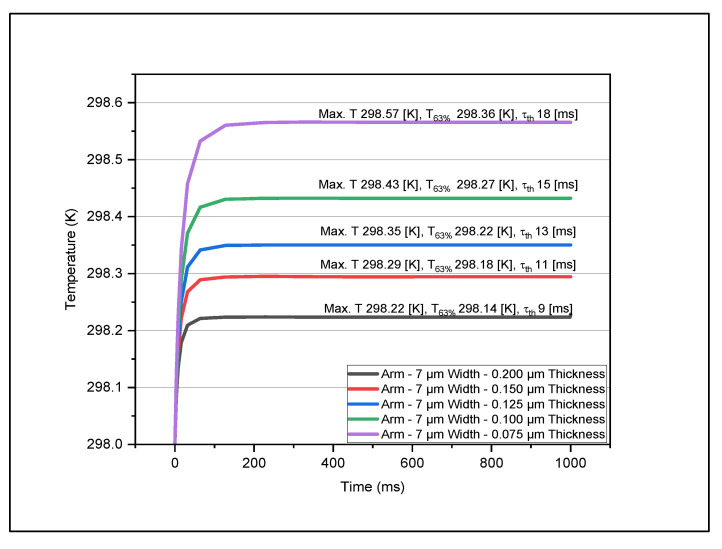
Variations in temperature with time at the sensing layer for different thicknesses with arm width kept fixed at 7 μm.

**Figure 6:**
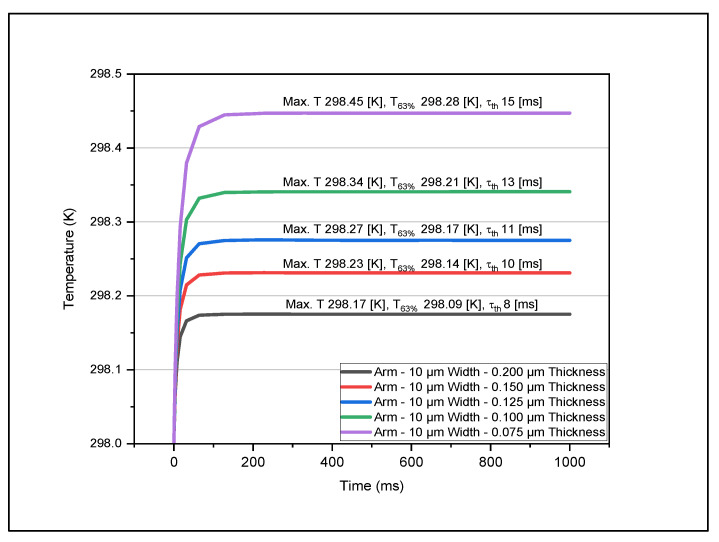
Variations in temperature with time at the sensing layer for different thicknesses with arm width kept fixed at 10 μm.

**Figure 7:**
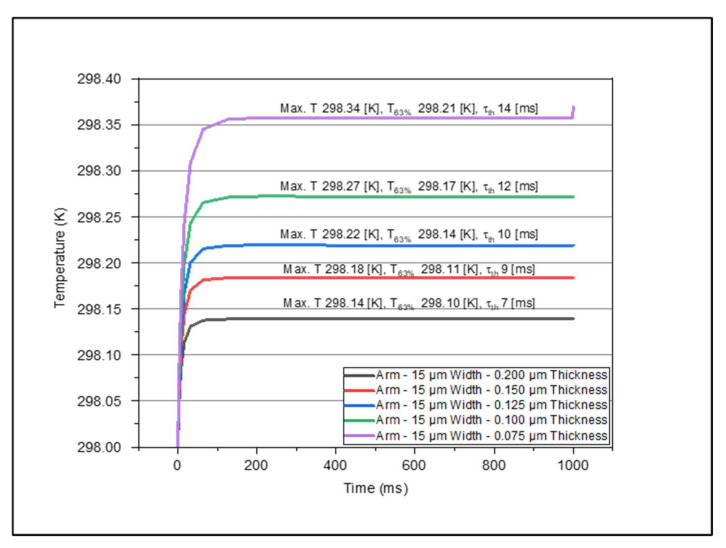
Variations in temperature with time at the sensing layer for different thicknesses with arm width kept fixed at 15 μm.

Figure 4, Figure 5, Figure 6 and Figure 7 show the data set of the heat transfer at the sensing layer for the different arm widths and thicknesses. In this graph, we indicate the maximum temperature (Max, T), 63% of the maximum temperature value ($T_{63\%}$), and the time constant ($\tau_{th}$). From this analysis, we found that as the thickness of various layers decreased, the temperature increased accordingly. The results are based on the dimensions of the various parts mentioned in Table 2. The optimum design had a sensing pixel size of 35 μm (length) by 35 μm (width), a thickness of 0.2 μm, an electrode arm thickness of 0.2 μm, and a width of 5 μm. The corresponding temperature increase on the sensing layer was 0.3 K. We obtained the maximum temperature in the sensing layer as we varied the width and thickness of the arm and contact layers. In Table 3, we recorded the maximum temperature value for each model. This table shows how the maximum temperature changed when we varied the thickness and the width. The maximum temperature rise was 298.8 K for an electrode arm thickness of 0.075 μm and width of 5 μm, and the lowest temperature rise was 298.14 K for an electrode arm thickness of 0.2 μm and width of 15 μm. We observe that the electrode arm designs with less thermal mass will have a higher temperature at the sensing layer, which means that they dissipate less thermal energy to the heat sink. Now, this dissipation capacity will determine the thermal time constant of the models when it reaches 63% of the maximum temperature. We described the resultant values of this analysis for various dimensions of electrode arms in Table 4. Then, to determine the thermal time constant related to the analytical value $T_{63\%}$, we graphically analyzed the results mentioned in Table 3 to determine the thermal time constant using Equation (14). These values are shown in Table 5.

From this analysis, we determined that the model with a lower thermal mass (5 μm width–0.075 μm thickness) has the highest thermal time constant, 24 ms. On the other hand, the model with the highest thermal mass has the lowest thermal time constant. The reason for this is that the maximum temperature of 5μm width–0.075 μm thickness model is five times greater than the maximum temperature of 15 μm width–0.2 μm thickness model. This result helps us address the question of what model would be the best option for an excellent thermal time response based on the temperature change. The lower the thermal time constant is, the higher the device speed would be. To improve the performance of the microbolometer, we need to balance the TCR of the sensing layer and the heat dissipation. This statement refers to the fact that we need a significant change in temperature in the sensing layer and a fast thermal time response. Therefore, the 5 μm width–0.075 μm thickness and 15 μm width–0.2 μm thickness models are not good options. Now we can consider the options of 5 μm width–0.2 μm thickness, which show a considerable temperature change in the sensing layer and a thermal time response of 11 ms, comparable with the DRS dual cavity microbolometer made in the base of VO_x_ [16]. Based on this conclusion, we consider this model, 5 μm width–0.2 μm thickness, for further analysis.

### 3.3. Electrical Potential

**Figure 8:**
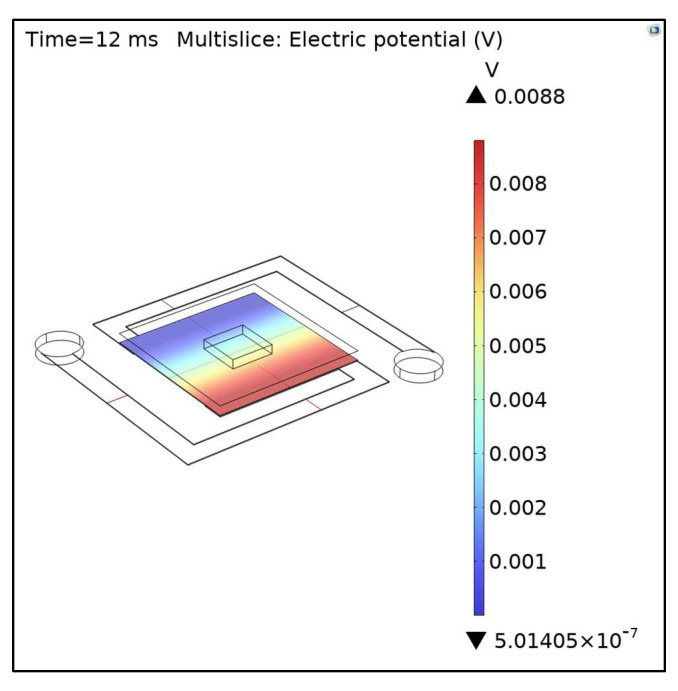
Visualization of electric potential at the sensing layer after 12 ms.

To calculate the responsivity and detectivity, we need to know the electrical response of the device. To simulate this phenomenon, we used the EIC. The microbolometer’s response was simulated based on the different electrical potentials. This difference in potential is related to the change in the semiconductor resistance because of the heat absorption. In Figure 8, we showed the electrical potential for the model of 5 μm width and 0.2 μm thickness. The ECI is coupled to the HTSI, making possible the analysis of the heat effect on the electrical properties of the device. Therefore, we varied the arm’s thickness and computed how the electrical potential was affected. In Table 6, we show the resultant change of the resistance, conductivity, and voltage for a bias current of 0.15 μA. The initial resistance, conductivity, and voltage of the sensing layer for the 5 μm width–0.200 μm thickness model were ~1.049 MΩ, 3.409 S/m, and 157.626 mV, respectively.

### 3.4. Responsivity and Detectivity

**Figure 9:**
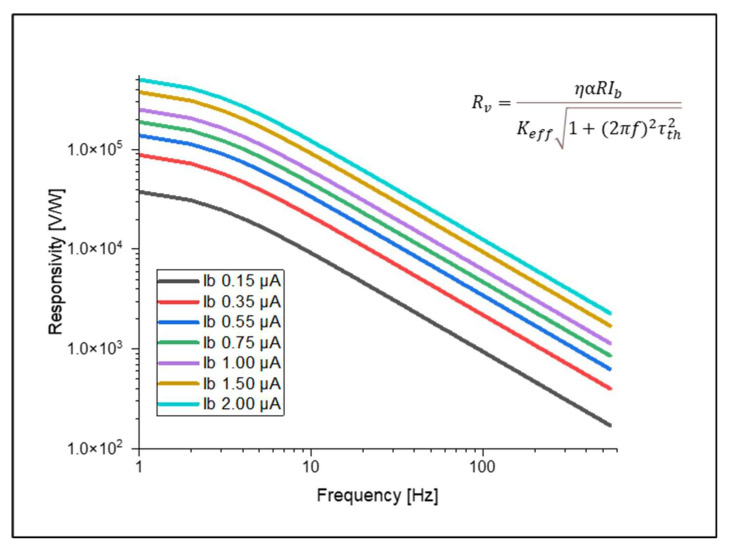
Variations in responsivity with different bias currents for the microbolometer with the 5 μm width and 0.2 μm thickness arms.

**Figure 10:**
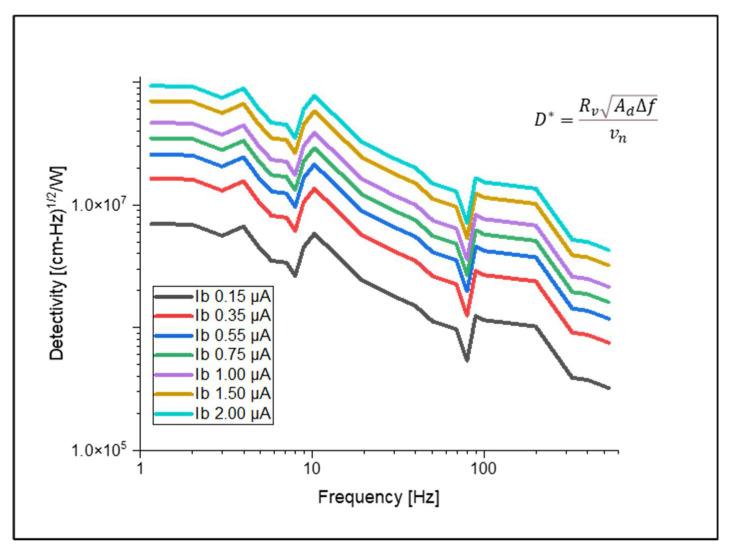
Variations in detectivity with bias currents for the microbolometer with the 5 μm width and 0.2 μm thickness arms.

The responsivity and detectivity were calculated analytically. For this, we used the results of thermal time response and electrical potential from the structure, which has a width of 5 μm and a thickness of 0.2 μm. These values were calculated for different bias currents. In Figure 9, we show the voltage responsivity, while in Figure 10, we show the detectivity at different bias current values. These results, compared with bolometers based on VO_x_, showed an improvement in the responsivity and detectivity [25,26,27,28,29,30,31,32,33]. The results of our work were compared with other work and mentioned in Table 7. Our detectivity value is lower than the others. The reason behind this is the relatively higher noise associated with the sensing layer we used (Ge_36_Si_0.04_Sn_11_O_43_) as compared to VO_x_. Our results are comparable with [8], which used COMSOL to design and simulate the microbolometers. The highest values of responsivity and detectivity we obtained were $5.040\times 10^{5}\;V/W$ and $9.357\;10^{7}\;cm-Hz^{1/2}/W$. For this, we considered a 2 μA bias current at 1 Hz.

## 4. Conclusions

After performing the simulations and evaluating the results, we can conclude that the model with an arm width of 5 μm and a thickness of 0.2 μm is the most promising for the next step of the project. This is because the model with those dimensions demonstrated a response time of 11 ms for a maximum heat change of 0.32 K. Although some models demonstrated a shorter response time, the temperature difference was insignificant. For example, the 15 μm arm width model with a thickness of 0.2 μm showed a response time of 7 ms, which is a good value for design, but the maximum temperature rise was only 0.09 K. The simulations showed that the relationship between the response time and the heat capacity of the sensing layer is directly conditioned by the effective conductivity of the model. Concluding, we can state that if we increase the arm’s cross-sectional area, referring to its width and thickness, the model in question will show a minimum change in temperature and, therefore, a shorter response time. Consequently, we consider models with a low arm cross-section area to obtain a significant change in temperature at the sensing layer and good time response, as shown by the model with an arms width of 5 μm and 0.2 μm thickness. The resultant values of this analysis were compared with others. Table 7 includes a comparison of the figures of merit of our work with others which are both simulations and experimental. The current work shows an improvement in the figures of merit of microbolometers. This improvement is related to the TCR value of the Ge_36_Si_0_._04_Sn_11_O_43_ and the parametrization of the double cavity model. The simulation work can be used to optimize the design of the microbolometer device, such as the thickness of the Ge_x_Si_y_Sn_z_O_r_ layer or the pixel size of the microbolometer array, to further improve its performance. Future work will involve the fabrication of the device using the micromachining fabrication process and comparing the experimental result with the simulation values.

## Figures and Tables

**Table 1:** Materials properties used for device design.

	Domains	Post, Arms, and Contacts	Sensing Layer	Absorbing Layer
	Materials	Ti	Ge_36_Si_0.04_Sn_11_O_43_	Ni_80_Cr_20_
Properties	Density	4.94 g/cm^3^	3.182 g/cm^3^	8.31 g/cm^3^
	Heat capacity at constant pressure	710 J/(kg∙K)	320 J/(kg∙K)	500 J/(kg∙K)
	Thermal conductivity	7.5 W/(m∙K)	59.9 W/(m∙K)	17 W/(m∙K)
	Relative permittivity	1	1	0.85
	Reference resistivity	4.7 × 10^−7^ Ωm	0.293317906 [Ω∙m]	n/a
	TCR	0.1 1/K	−0.0366 1/K	n/a

**Table 2:** Various layers of microbolometers with their dimensions.

Parts	Materials	Dimensions		
		Length [μm]	Width [μm]	Thickness [μm]
Absorbing layer	Ni_80_Cr_20_	35	35	0.2
Post	Ti	10	10	2.2
Sensing layer	Ge_0.36_Si_0.04_Sn_11_O_43_	35	35	0.2
Arm	Ti	132.5, 138.5, 143.5, 165.5	5, 7, 10, 15	0.2, 0.15, 0.125, 0.075
Contact	Ti	10, 14, 20, 30	10, 14, 20, 30	2.2

**Table 3:** Maximum temperature variations in the absorber layer with width and thickness of electrode arm.

w [μm]	t [μm]	Max. T [K]	t [μm]	Max. T [K]	t [μm]	Max. T [K]	t [μm]	Max. T [K]	t [μm]	Max. T [K]
5	0.2	298.32	0.15	298.42	0.125	298.50	0.1	298.62	0.075	298.80
7	0.2	298.22	0.15	298.29	0.125	298.35	0.1	298.43	0.075	298.57
10	0.2	298.17	0.15	298.23	0.125	298.27	0.1	298.34	0.075	298.45
15	0.2	298.14	0.15	298.18	0.125	298.22	0.1	298.27	0.075	298.34

**Table 4:** Sixty-three percent of the maximum temperature variation in the absorber layer with width and thickness of electrode arm.

w [μm]	t [μm]	T_63%_ [K]	t [μm]	T_63%_ [K]	t [μm]	T_63%_ [K]	t [μm]	T_63%_ [K]	t [μm]	T_63%_ [K]
5	0.2	298.20	0.15	298.26	0.125	298.31	0.1	298.40	0.075	298.50
7	0.2	298.14	0.15	298.18	0.125	298.22	0.1	298.27	0.075	298.36
10	0.2	298.10	0.15	298.14	0.125	298.17	0.1	298.21	0.075	298.28
15	0.2	298.09	0.15	298.11	0.125	298.14	0.1	298.17	0.075	298.21

**w**—width; **t**—thickness; **T_63%_**—63% of the maximum temperature.

**Table 5:** Variation in time constants for various widths and thicknesses of electrode arm layer.

w [μm]	t [μm]	$\tau_{th}$ [ms]	t [μm]	$\tau_{th}$ [ms]	t [μm]	$\tau_{th}$ [ms]	t [μm]	$\tau_{th}$ [ms]	t [μm]	$\tau_{th}$ [ms]
5	0.2	11	0.15	14	0.125	16	0.1	19	0.075	24
7	0.2	9	0.15	11	0.125	13	0.1	15	0.075	18
10	0.2	8	0.15	10	0.125	11	0.1	13	0.075	15
15	0.2	7	0.15	9	0.125	10	0.1	12	0.075	14

**w**—width; **t**—thickness; $\tau_{th}$—thermal time constant.

**Table 6:** Resultant change in the resistance, conductivity, and voltage for a bias current of 0.15 μA.

Model	Δ Resistance [MΩ]	Δ Conductivity [S/m]	Δ Voltage [mV]
5 μm w–0.200 μm t	0.01232	0.04059	1.8512
5 μm w–0.150 μm t	0.01622	0.05354	2.4349
5 μm w–0.125 μm t	0.01921	0.06362	2.8855
5 μm w–0.100 μm t	0.02365	0.07862	3.5515
5 μm w–0.075 μm t	0.02984	0.10061	4.4784

**w**—width; **t**—thickness.

**Table 7:** Comparison of figures of merit for various microbolometer design.

$K_{eff}$ (W/K)	$\tau_{th}$ (ms)	$R_{v}$ (V/W)	D* (cmHz^1/2^/W)	Dimension (μm)	Refs.	Category
$1.0135\times 10^{-7}$	11	$5.04\times 10^{5}$	$9.357\times 10^{7}$	35×35	Current work	Simulation
-	-	$8.69\times 10^{6}$	-	35×35	[25]	Experimental
-	-	$8.9\times 10^{5}$	$2.2\times 10^{9}$	65×60	[26]	Experimental
-	-	$1.24\times 10^{4}$	$5.86\times 10^{8}$	24×26	[27]	Experimental
-	-	$5\times 10^{2}$	$1\times 10^{15}$	−	[28]	Simulation
-	-	$5.91\times 10^{4}$	$2.34\times 10^{9}$	40×40	[29]	Simulation
$1.556\times 10^{-7}$	10.35	$1.4\times 10^{4}$	$2.31\times 10^{9}$	25×25	[30]	Experimental
-	-	$2.0\times 10^{4}$	$2\times 10^{7}$	50×50	[31]	Simulation
$3.08\times 10^{-7}$	-	$2.13\times 10^{4}$	$2.33\times 10^{9}$	40×40	[32]	Experimental
-	10	$1.4\times 10^{4}$	$2.1\times 10^{8}$	32×32	[33]	Experimental

## Data Availability

The data is available if requested.
